# Fibrinogen function indexes are potential biomarkers for evaluating the occurrence and severity of diabetic foot

**DOI:** 10.1186/s13098-022-00960-4

**Published:** 2022-12-02

**Authors:** Jinying Zhang, Jiayu Lin, Bo Liang, Lijun Chen, Xinna Yang, Mimi Li

**Affiliations:** 1 Department of Neurology, The Second Affiliated Hospital of Fujian Medical University, Quanzhou, 362000 Fujian China; 2 Department of Endocrinology, The Second Affiliated Hospital of Fujian Medical University, No. 950 Donghai Street, Fengze District, Quanzhou, 362000 Fujian China

**Keywords:** Diabetic foot, Wagner classification, Angle α, K value, Fibrinogen

## Abstract

**Background and objectives:**

Research suggests that fibrinogen (Fib) concentrations are used to assess the occurrence and severity of diabetic foot (DF) and to monitor the progression of diabetic foot in patients. However, its correlation with Fib function has not been reported. Here, angle α and k value, reflecting the Fib function, were used to analyse its correlation with DF, and their potential as biological indicators for evaluating the occurrence and severity of DF was explored.

**Subjects and methods:**

This clinical study enrolled 163 type 2 diabetes mellitus (T2DM) patients, who were divided into the diabetes with DF (84 cases) group, diabetes with no DF (79 cases) group. Meanwhile, 90 healthy unrelated subjects were enrolled as controls.

**Results:**

Angle α and fibrinogen levels increased greatly in subjects with DF compared with those without. The k value levels greatly decreased in subjects with DF compared with those without (P < 0.01). Spearman correlation analysis showed that angle α and fibrinogen were positively correlated with DF grading (r = 0.635, P < 0.01; r = 0.616, P < 0.01), k value was negatively correlated with DF (r= − 0.589, P < 0.01). ROC curve analysis showed that the optimal cut-off point for angle α to distinguish patients with DF from those without was 62.85 deg, with a sensitivity of 78.6% and specificity of 78.7%. The optimal cut-off point for k value was 1.75 min, with a sensitivity of 82.1% and specificity of 65.8%. The optimal cut-off point for fibrinogen was 3.85 g/l, with a sensitivity of 63.1% and specificity of 98.2%. The optimal cut-off point for angle α to evaluate the risk of diabetic foot progression was 70.20 deg, with a sensitivity of 73.2% and specificity of 90.7%. The optimal cut-off point for k value was 1.25 min, with a sensitivity of 67.9% and specificity of 90.8%. The optimal cut-off point for fibrinogen was 4.12 g/l, with a sensitivity of 85.7% and specificity of 93.5%.

**Conclusion:**

Angle α, k-value and fibrinogen have clinical significance on the risk of occurrence and development of diabetic foot, which can contribute to early diagnosis and early clinical intervention in DF.

Diabetic foot (DF) is a serious diabetic complication that refers to the destruction of the skin and deep tissues (including muscle and bone) distal to the ankle joint, often combined with arterial occlusion and infection of the lower extremity [1]. The global prevalence of diabetic foot is 6.3%, and the prevalence of diabetic foot in China is about 4.1% [2]. A recent research reported that the 1-, 2-, and 5-year survival rates for diabetic foot disease were 81%, 69%, and 29%, respectively [3]. The pathogenesis of DF is complex and is usually associated with micro-vascular and macro-vascular alterations. A large clinical study found that peripheral vascular disease (PAD) in patients with T2DM is a serious complication. A large clinical study found that the prevalence of PAD in patients with T2DM was 23.5% [2], and diabetic patients with combined PAD are more likely to develop ulceration and gangrene of the limb, significantly increasing the risk of amputation. Therefore, finding indicators that predict the risk of occurrence and progression of diabetic foot and early intervention can help improve the quality of life.

The International Working Group on the Diabetic Foot has developed guidelines for the diagnosis of the diabetic foot. However, most of the relevant studies have focused on the analysis of risk factors associated with the diabetic foot and comprehensive management. There is a lack of uniform quantitative standards for biomarkers that predict the risk of diabetic foot occurrence and progression. In recent years, research suggests that haemodynamic disorders are involved in the pathogenesis of DF. The occurrence of diabetic foot is closely related to micro-angiopathy, micro-thrombosis in the lower limb, which may predate the presentation of diabetic foot. The inflammatory response, microcirculation disorders and hypercoagulability promote the occurrence and development of DF. Lower limb vasculopathy has been found to be closely related to abnormal coagulation activity [3]. Fibrinogen is an important determinant of blood viscosity and platelet aggregation [4, 5] and may play a role in endothelial injury [6], low-osmolar fibrin clot formation [7], thrombosis [8], blood flow abnormalities [9] and platelet overactivity [10]. These studies suggest that fibrinogen is significantly associated with vascular lesions and thrombosis. Fib is closely related to DF.

Thromboelastography (TEG) assesses the human coagulation system, providing information on platelet function, coagulation and fibrinolysis. The time point between the placement of venous blood into the TEG analyzer and the formation of the first fibrin clot (tracing amplitude up to 2 mm) is used as the starting time point. The angle α is the angle between the tangent line and the horizontal line from the point of clot formation to the arc of the maximum curve. The k value is the time required between this time point and the tracing amplitude of 20 mm, reflecting the rate of clot formation. An increase in angle α and a decrease in k-value can be an important observational indicator for the development of vascular lesions [11]. The angle α and k value reflect the rate of clot formation and fibrinogen function, which may act as novel biomarkers of DF. However, to date, there has been no report on the relationship between Fib function and DF. In this study, we analyzed the relationship between angle α, k value, fibrinogen and diabetic foot, aiming to investigate the optimal cut-off point value of the above three index tests on the risk of occurrence and progression of diabetic foot, and to provide a reference basis for clinical work.

## Research design and method

### General information

163 type 2 DM patients who were hospitalized in the Department of Endocrinology of the Second Affiliated Hospital of Fujian Medical University from March 2020 to December 2021 were included in this study at all, including 84 patients with DF (DF group) and 79 patients without DF (NDF group). 90 healthy control subjects (NC group) registered with our hospital physical examination centre were randomly enrolled in the study. The criteria for inclusion were as follows: (1) all participating patients met the type 2 DM diagnostic criteria issued by the American Diabetes Association (ADA) in 2012 [12] and the DF diagnostic criteria issued by The Wagner classification system [13], and patients with a Wagner grade < 3 were defined as mild DF, while patients with a Wagner grade ≥ 3 were defined as severe DF. The exclusion criteria were the patients with type 1 diabetes, gestational diabetes and secondary diabetes, patients with acute complications of combined diabetes (such as diabetic ketoacidosis, non-ketotic hyperosmolar state), patients with various other acute and chronic infections, trauma and surgery, patients with combined cardiac, hepatic and renal insufficiency, arterial and venous embolism and cerebrovascular events, patients with rheumatic immune diseases, hematological diseases and tumors, patients with use of drugs that have an impact on coagulation function (such as exogenous fibrinogen, hormones, antiplatelet agents, anticoagulants, etc.). This study was approved by the hospital and university scientific and ethics committees, and each patient was included in the study signed informed consent.

## Clinical feature

Demographic data (gender, age), body mass index (BMI), duration, drugs used related to diabetes, localization of wound, depth of wound, and presence of purulent discharge were recorded during admittance. Blood samples were taken after 10–12 h of overnight fasting, and fasting blood glucose (FBG), glycosylated hemoglobin (HbA1c), blood lipids, fibrinogen levels, angle α and k value were studied. The angle α and k value were assessed by a TEG analyser (LBPU-8800). Fibrinogen was measured by using a coagulometer device (Starco STAR-MAX). Fasting plasma glucose, blood lipids were measured by Biochemistry (Roche C702). HbA1c measurements were performed by Tosoh (HLC-723G8). All tests were were studied in the biochemistry laboratory of our hospital and performed in a blinded manner.

### Statistical analysis

SPSS (Statistical Product and Service Solutions) 26.0 software was used for the statistical analysis. The data of continuous variables obeying normal distribution were expressed by“mean ± standard deviation (x ± s)”. One-way ANOVA test was used for the comparisons of the groups three with normal distribution, and the LSD method was used for multiple comparisons. A *t*-test was used for comparison between the two groups. The relation of the angle α, k value and fibrinogen levels to the DF was calculated using Spearman’s correlation analysis. Receiver operating characteristic (ROC) analysis was used to obtain the optimal cut-off point for predicting the risk of diabetic foot occurrence and progression by area under the curve (AUC) for angle α, k value, and fibrinogen. Significance was evaluated at a level of p < 0 05.

## Results

**Fig. 1:**
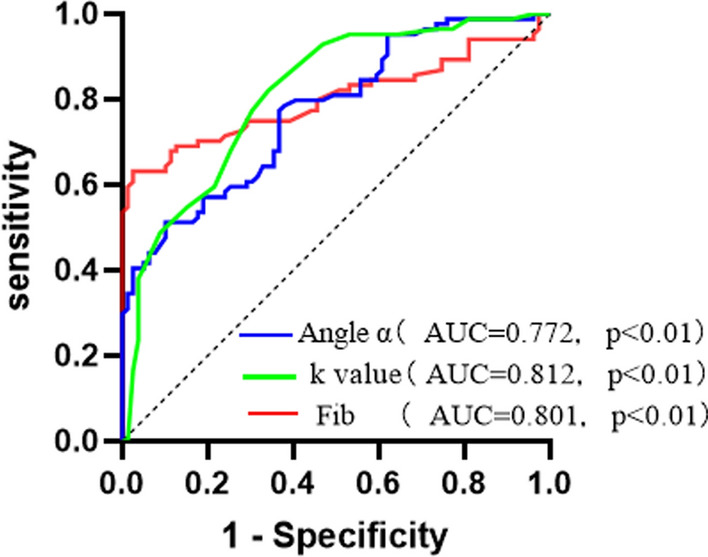
ROC curves for the evaluation of risk of DF using angle α, k value and Fib

**Fig. 2:**
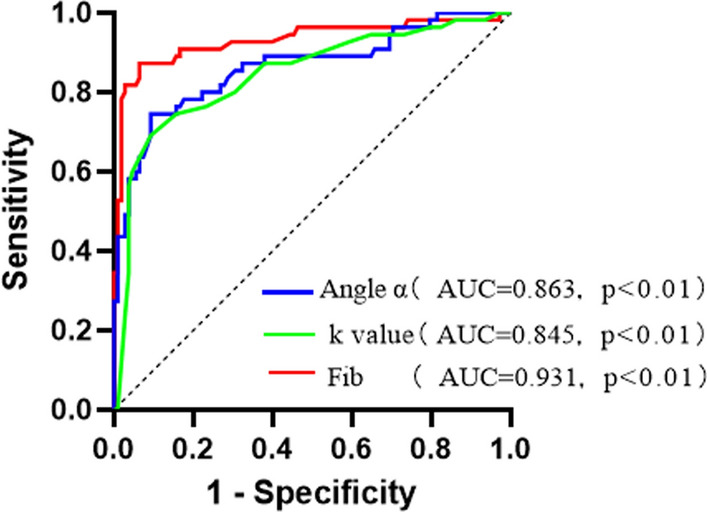
ROC curves for the evaluation of severity of DF using angle α, k value and Fib

The study was completed by 253 subjects, including 90 healthy control subjects, 84 diabetic subjects with DF and 79 diabetic subjects without DF (Table 1). Diabetic foot group was further staged into mild DF (29 cases) group and severe DF (55 cases) group (Table 2). Among the three groups of subjects,there were no differences between any two groups in the following variables: gender, age, BMI, blood lipids (cholesterol, triglyceride, low-density lipoprotein cholesterol (LDL-C), high-density lipoprotein cholesterol (HDL-C)). The duration of disease was longer in the DF group than in the non-DF group. Fasting glucose (P < 0.05) and HbA1c (P < 0.01) were higher in the DF group than in the NDF group, and the differences were statistically significant. Diabetic subjects with DF showed increased levels of angle α and Fib, and decreased levels of k value compared with patients without DF and control group (P < 0.01). Plasma α-angle and fibrinogen levels were significantly higher in patients with severe DF than in patients with mild DF, and k value levels were significantly lower than in patients with mild DF, with statistically significant differences (P < 0.01) (Table 2). Fib and angle α levels were positively correlated with diabetic foot grading (r = 0.635, P < 0.01; r = 0.616, P < 0.01), k value was negatively correlated with DF (r= − 0.589, P < 0.01). Correspondingly, Fib levels was positively correlated with angle α and negatively correlated with k value (r = 0.553, P < 0.01, r=-0.526, P < 0.01). ROC curve analysis showed that the optimal cut-off point for angle α to distinguish patients with DF from those without was 62.85 deg, with a sensitivity of 78.6% and specificity of 78.7%, and the highest AUC equal to 0.772(P < 0.001). The optimal cut-off point for k value was 1.75 min, with a sensitivity of 82.1% and specificity of 65.8%, and the highest AUC equal to 0.812(P < 0.001). The optimal cut-off point for fibrinogen was 3.85 g/l, with a sensitivity of 63.1% and specificity of 98.2%, and the highest AUC equal to 0.801 (P < 0.001). The optimal cut-off point for angle α to evaluate the risk of diabetic foot progression was 70.20 deg, with a sensitivity of 73.2% and specificity of 90.7%, and the highest AUC equal to 0.863 (P < 0.001). The optimal cut-off point for k value was 1.25 min, with a sensitivity of 67.9% and specificity of 90.8%, and the highest AUC equal to 0.845 (P < 0.001). The optimal cut-off point for fibrinogen was 4.12 g/l, with a sensitivity of 85.7% and specificity of 93.5%, and the highest AUC equal to 0.931 (P < 0.001) (Figs. 1 and 2).

**Table 1:** Comparison of clinical features and laboratory parameters between different groups

Group	NC group	NDF group	DF group
Case (male/female)	48/42	51/28	53/31
Age (years)	57.5 ± 8.4	58.0 ± 9.5	60.1 ± 9.7
Duration (years)	–	5.5 ± 5.3*	7.3 ± 6.2*^#^
BMI(kg/m^2^)	21.6 ± 2.0	21.0 ± 2.1	22.1 ± 2.3
FBG(mmol/l)	5.11 ± 0.49	9.46 ± 4.14*	10.74 ± 4.58*^#^
HbA1c(%)	5.6 ± 0.4	8.2 ± 2.2*	9.8 ± 2.6*^☆^
TC(mmol/L)	4.57 ± 1.37	4.85 ± 1.20	4.86 ± 1.23
TG(mmol/L)	1.74 ± 1.13	1.77 ± 1.33	1.73 ± 1.30
LDL–C(mmol/L)	2.21 ± 0.94	1.91 ± 1.02	1.95 ± 1.36
HDL–C(mmol/L)	1.24 ± 0.36	1.16 ± 0.45	1.25 ± 0.52
Fib(g/L)	2.82 ± 0.57	3.04 ± 0.52	4.94 ± 2.01*^☆^
Angle α(deg)	58.8 ± 5.1	61.4 ± 7.7^△^	69.5 ± 7.3*^☆^
k value(min)	2.1 ± 0.4	1.9 ± 0.5*	1.3 ± 0.4*^☆^

(vs. NC group, ^△^P < 0.05, *P < 0.01; vs. NDF group, ^#^P < 0.05, ^☆^P < 0.01)

**Table 2:** Comparison of Angle α, K value and fibrinogen in patients with different degrees of diabetic foot

Group	Mild DF group	Severe DF group
Case	29	55
Fib(g/L)	3.26 ± 0.96	5.82 ± 1.86^*^
Angle α(deg)	63.7 ± 5.5	72.5 ± 6.4^*^
k value(min)	1.6 ± 0.3	1.2 ± 0.4^*^

(vs. Mild DF group, ^*^P < 0.01)

## Discussion

The results of this study showed that angle α and k value, reflecting the Fib function, were potential biological indicators for evaluating the occurrence and severity of DF. In this study, patients with DF had higher angle α and Fib levels than diabetic patients without DF, and the levels of k value in patients with DF were significantly lower than those in diabetic patients without DF. More importantly, the angle α, Fib and k value were changed in the early stage of diabetic foot.

Diabetes mellitus, characterized by fasting hyperglycemia, is a risk factor for atherosclerotic thrombosis, and the diabetic foot is one of its serious chronic complications and a major cause of hospitalization and amputation in diabetic patients. Common risk factors that predispose to diabetic foot include poor glycemic control, peripheral neuropathy, and PAD. Domestic studies have shown that 19.5% of diabetic patients over 50 years of age and 35.4% of diabetic patients over 60 years of age have lower limb arterial lesions in China [1, 2]. In addition, the severity of the diabetic foot is associated with a higher rate of lower limb amputation, with 85% of DF patients progressing to low distal amputation. It has a high rate of disability and mortality, which seriously affects patients’ life and quality of life due to its psychological and social consequences.

The essence of lower extremity vasculopathy in the diabetic foot is atherosclerosis. Platelet hyperreactivity, coagulation status and abnormal fibrinolytic function are prevalent in patients with diabetic foot and worsen with the progression of the disease [14]. Prostacyclin and nitric oxide produced by normal endothelial cells have anti-platelet aggregation and adhesion functions, and metabolic abnormalities such as sustained elevation of blood glucose cause impaired endothelial function, inhibition of endothelial nitric oxide synthase activity, decreased nitric oxide release, and enhanced platelet adhesion and aggregation [15]. At the same time, metabolic abnormalities increase fibrinogen activator inhibitor and fibrinogen, causing a hypercoagulable state of blood in diabetic foot patients, which predisposes to thrombus formation and causes microvascular and lower extremity macroangiopathy [16]. It would be significant to predict the occurrence and development of diabetic foot in advance and intervene early when peripheral vasculopathy is present in diabetic patients but before diabetic foot complications develop.

Fibrinogen, synthesized mainly by hepatocytes, is the most abundant procoagulant factor in plasma. Fib is involved in atherosclerosis and thrombosis, reflects inflammatory changes and endothelial dysfunction in vascular lesions, contributes to a hypercoagulable state of blood, and is one of the underlying conditions for thrombosis and subclinical atherosclerosis [17]. Some studies have used fibrinogen for predicting diabetic foot and assessing the severity of diabetic foot, and its optimal cut-off points for determining the risk and severity of DF were 3.88 g/L and 4.74 g/L, respectively, with an area under the curve of 0.86 (sensitivity of 0.74, specificity of 0.87) and 0.73 (sensitivity of 0.76, specificity of 0.58) [18]. In the present study, we found that fibrinogen levels were significantly higher in patients with diabetic foot compared to those without diabetic foot, and the optimal cut-off points for predicting the occurrence and severity of DF were 3.85 g/L and 4.12 g/L, which were similar to the results of previous studies, further corroborating that Fib may be involved in the occurrence and development of diabetic foot as an important factor. Meanwhile, this study further confirmed that Fib was positively correlated with diabetic foot grading (r = 0.616), and the higher the Fib level, the higher the Wagner grading of diabetic foot, suggesting the need for timely clinical control of Fib levels to reduce or delay the occurrence and progression of diabetic foot.

TEG is a coagulation test technology that provides comprehensive testing of coagulation, fibrinolytic composition, and platelet function [19]. TEG has been used in the 1980 s for clinical applications such as immediate coagulation monitoring in a variety of conditions, predicting the risk of venous thrombosis, and guiding clinical component transfusion [20]. The angle α and k value represent the clot formation rate and reflect the Fib functional status. A decreased k value with an increased angle α indicates a high Fib level (hypercoagulation), while an increased k value with a decreased angle α indicates a low fibrinogen level (hypocoagulation). Several studies [21, 22] have found that increased angle α and decreased k value can be important observational indicators for the development of micro-vascular and macro-vascular lesions. In this study, we found that the best cut points for diagnosing diabetic foot were 62.85 deg (angle α 53-72 deg) and 1.75 min (k value 1-3 min), and the definition of the best cut point suggested that when angle α is higher than 62.85 deg and k value below 1.75 min alerted to the occurrence of diabetic foot. The sensitivity of angle α and k value for diabetic foot diagnosis was 78.6% and 82.1%, which was higher than that of fibrinogen for diabetic foot diagnosis. It suggests that TEG may be more sensitive than Fib test for earlier diagnosis of diabetic foot and thus early intervention. The present study also found that angle α and k value correlated with Wagner grade and Fib in patients with diabetic foot. Higher Wagner grade and higher fibrinogen content were associated with larger angle α and lower k value. The optimal cut-off point for angle α and k value to indicate a poor prognosis for patient with DF was 70.20 deg and 1.25 min. When monitoring for abnormalities in the above indicators, the addition of drugs that reduce blood hypercoagulation may have a beneficial effect on preventing and delaying the onset and progression of diabetic foot.

## Conclusion

This study further confirmed that hypercoagulable state and thrombosis may lead to the occurrence and development of diabetic foot. The Fib, angle α, and k value serve as indicators of coagulation function and may serve as potential biomarkers for the occurrence and severity of diabetic foot. Physicians can monitor Fib, angle α and the k value to detect diabetic foot in time. However, this study still has some limitations, and whether active interventions can significantly reduce the occurrence of adverse prognosis remains to be further studied. Moreover, the sample size of this study was not large, and further confirmation by a comprehensive study with a large sample is needed.

## Data Availability

The datasets used or analysed during the current study are available from the corresponding author on reasonable request.
